# Ionotropic Gelation Fronts in Sodium Carboxymethyl Cellulose for Hydrogel Particle Formation

**DOI:** 10.3390/gels7020044

**Published:** 2021-04-12

**Authors:** William N. Sharratt, Carlos G. Lopez, Miriam Sarkis, Gunjan Tyagi, Róisín O’Connell, Sarah E. Rogers, João T. Cabral

**Affiliations:** 1 Department of Chemical Engineering, Imperial College London, London SW7 2AZ, UK; miriam.sarkis16@imperial.ac.uk (M.S.); g.tyagi@imperial.ac.uk (G.T.); r.oconnell17@imperial.ac.uk (R.O.); 2 Institute of Physical Chemistry, RWTH Aachen University, Landoltweg 2, 52056 Aachen, Germany; cglopez@cantab.net; 3 ISIS, Rutherford Appleton Laboratory, Harwell, Didcot OX11 0QX, UK; sarah.rogers@stfc.ac.uk

**Keywords:** polyelectrolyte gel, sodium carboxymethyl cellulose, microparticles, hydrogel, small angle neutron scattering, ionic gelation, front propagation, FTIR

## Abstract

Hydrogel microparticles (HMPs) find numerous practical applications, ranging from drug delivery to tissue engineering. Designing HMPs from the molecular to macroscopic scales is required to exploit their full potential as functional materials. Here, we explore the gelation of sodium carboxymethyl cellulose (NaCMC), a model anionic polyelectrolyte, with Fe^3+^ cations in water. Gelation front kinetics are first established using 1D microfluidic experiments, and effective diffusive coefficients are found to increase with Fe^3+^ concentration and decrease with NaCMC concentrations. We use Fourier Transform Infrared Spectroscopy (FTIR) to elucidate the Fe^3+^-NaCMC gelation mechanism and small angle neutron scattering (SANS) to spatio-temporally resolve the solution-to-network structure during front propagation. We find that the polyelectrolyte chain cross-section remains largely unperturbed by gelation and identify three hierarchical structural features at larger length scales. Equipped with the understanding of gelation mechanism and kinetics, using microfluidics, we illustrate the fabrication of range of HMP particles with prescribed morphologies.

## 1. Introduction

Hydrogel microparticles (HMPs), often referred to as “microgels”, are of great interest for their applications in cell and drug encapsulation and delivery [1], tissue engineering [2] and food products [3]. Preparation of HMPs involves the generation of polymer-containing droplets, in bulk or microfluidics, followed by solidification induced by covalent crosslinking (e.g., thiol-ene click chemistry or radical polymerisation [4,5,6]), or physical crosslinking with added salt (e.g., addition of CaCl_2_ to sodium alginate [7]).

Ionic crosslinking of polyelectrolytes, including many biopolymers, is a facile approach that relies on the interaction of the polyelectrolyte functional groups (e.g., carboxylate) with oppositely charged ions. The size, valency and hydration of added ion, and interaction type play an important role in the phase behaviour [8], crosslinking and rheological properties of polyelectrolytes [9,10,11]. Specific ions can induce lateral aggregation and crosslinking of polyelectrolyte solutions, resulting in a solid precipitate or gel formation [12,13,14,15,16]; salt addition can also alter the thermodynamics of mixing of the solution and induce considerable volume changes in gels without significantly altering their cross-link density [17]. Numerous other mechanisms, including phase separation, have been shown to underpin gelation processes [18]. A combination of osmotic, rheological, spectrosocpic and scattering measurements are generally required to examine the nature of the specific “gelation” process at play. When these reactions are contained within 10–1000 μm droplets, HMPs form. The generation of monodisperse droplets with prescribed dimensions can be readily achieved in microfluidics and employed by several groups to fabricate HMPs (e.g., [7,19,20,21]). However, a multiscale understanding of HMP formation and a predictive ability to design particle structure and performance remains elusive.

**Figure 1:**
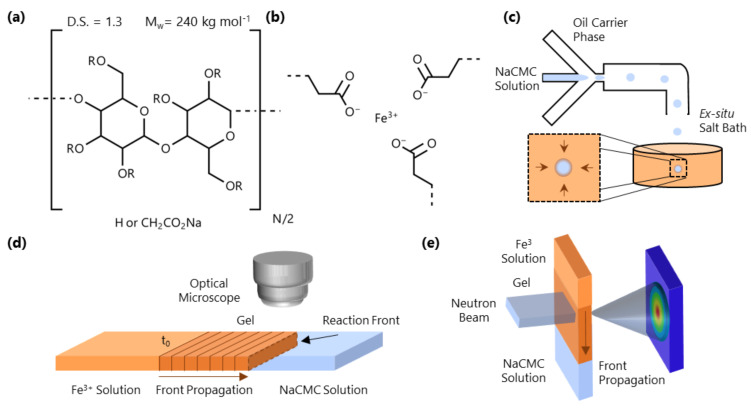
(**a**) Monomer structure of NaCMC; the polymer investigated has $M_{w}≃$ 240 kg mol^−1^ (PDI ≃ 3–4 [30,39]) and degree of substitution (D.S.) = 1.3, where D.S. is the number of carboxymethyl groups substituted at the hydroxyl position (R) of the cellulose monomer. (**b**) Schematic of the ionotropic gelation induced by Fe^3+^. (**c**) NaCMC/H_2_O droplet formation in microfluidics (in oleic acid) and ex-situ microparticle formation in an aqueous Fe^3+^ solution bath. (**d**) Schematic of a 1D front kinetics experiment in a microcapillary, tracking the evolution of the gelation interface over time. The experiment is initiated at the initial fluid-fluid interface at time $t_{0}$. (**e**) Spatio-temporal SANS experiment employing a horizontal slit beam resolving the molecular-scale conformational changes accompanying gelation.

Here, we report the microfluidic preparation of HMPs from sodium carboxymethyl cellulose (NaCMC) and investigate the underpinning gelation mechanism and kinetics with a multivalent cation. NaCMC is a semiflexible, anionic, water-soluble polyelectrolyte and one of the most widely used cellulose derivatives, with applications including food [22,23], enhanced oil recovery [24,25], medical treatments [26] and drug delivery [27]. NaCMC with a degree of substitution (D.S.) > 1, characterising the number of hydroxyl groups on the cellulose backbone substituted (out of a maximum of 3), is molecularly soluble in salt-free solutions [28] (see Figure 1a). Upon the addition of monovalent salts (e.g., Na^+^), electrostatic interactions between NaCMC chains are screened and the polyelectrolyte behaves analogously to a neutral polymer [29,30]. Addition of multivalent ions to NaCMC solutions can also solely screen electrostatics (e.g., Mg^2+^) and can cause aggregation (e.g., Ca^2+^, Ba^2+^, Zn^2+^) and even precipitation [31]. Solutions of CMC with divalent counterions display higher entanglement densities than those of NaCMC, but no gel formation formation is observed [11]. Trivalent ions (e.g., Al^3+^ or Fe^3+^) have been reported to cause gelation [32,33,34], rationalized in terms of the crosslinking of multiple carboxylate functionalities off the cellulose backbone as illustrated in Figure 1b. The sol-gel transition was confirmed by rheological measurements made by Ishii et al. [35] on NaCMC solutions with different concentrations of added Al^3+^ ions.

In this paper, we examine the gelation of salt-free NaCMC solutions with trivalent iron cations (Fe^3+^), which is then employed for formation of HMPs. Figure 1c depicts an example of how microfluidics can be used to generate NaCMC HMPs by droplet immersion into an Fe^3+^-containing bath. We first establish, using microfluidic channels (Figure 1d), that the gelation process can be described in terms of a reaction-diffusion front, often seen in thermodynamically driven ordering processes [36,37,38], which originates at the interface of the polyelectrolyte and salt solutions. We track the kinetics of the 1D front propagation as a function of both NaCMC (8.5–43 g/L) and Fe^3+^ (0.06–0.68 M) concentration. We then examine the molecular-scale interactions of Fe^3+^ with the polyelectrolyte via Fourier Transform Infrared Spectroscopy (FTIR) and investigate the spatio-temporal front propagation at the nanoscale employing small-angle neutron scattering (SANS), as depicted in Figure 1e. We employ time-resolved SANS to profile the travelling wave during front propagation, and then under asymptotic quasi-static conditions and spatially resolve the gel structure. Finally, we illustrate a range of HMP morphologies that can be generated with this approach.

## 2. Results and Discussion

### 2.1. Gelation Front Kinetics

**Figure 2:**
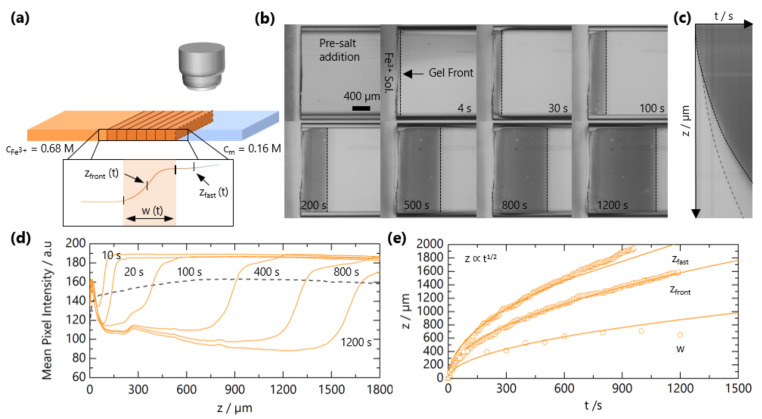
Experimental determination of NaCMC-FeCl_3_ gelation kinetics. (**a**) Schematic of the microchannel experimental setup to track the travelling gel fronts. Inset depicts a representative optical intensity profile for a gel front; front position $z_{front}\left(t\right)$, width w(t) and fast front position $z_{fast}\left(t\right)$. (**b**) Optical micrographs of gel front propagation at selected times. (**c**) Horizontal slice from (**b**) as a function of time yields $z_{front}\left(t\right)$. The solid line traces $z_{front}\left(t\right)$, and the dashed line traces the leading front $z_{fast}\left(t\right)$, which is difficult to observe by the naked eye. (**d**) Mean pixel intensity of reflected light as a function of position along microchannel *z* for different times. (**e**) Extracted $z_{front}\left(t\right)$, $z_{fast}\left(t\right)$ and w(t) as a function of time. Lines are fits to $z={\left(D_{f}t\right)}^{1/2}$, where $D_{f}$ is a descriptive 1D diffusion coefficient.

The experimental setup for tracking the front propagation of gels using optical microscopy is depicted schematically in Figure 2a. Figure 2b shows representative images as the front progresses for solutions with *c* = 0.16 M and $c_{S}=0.68$ M and a horizontal slice of the time-resolved images in Figure 2c. The integrated intensity as a function of the z coordinate (along the microchannel length) is presented in Figure 2d. By optical microscopy, the front appears “sharp”, and closer inspection enables the quantification of a front width *w* (∼few 100 μm); in addition, the front position $z_{front}\left(t\right)$, a precursor “fast front” $z_{fast}\left(t\right)$, is found to travel ahead of $z_{front}\left(t\right)$. We can thus identify two regions during gel propagation: $z_{fast}\left(t\right)$ corresponds the leading diffusion front of Fe^3+^ ions into the solution of NaCMC, where the concentration is not sufficiently high to form an opaque gel but provides optical contrast. NaCMC gels are known to be stable to syneresis [40]. We note that the hydrolysis of Fe^3+^ generates other equilibrium ionic species thatcould also interact with NaCMC [41]; these can, however, be estimated to form in low concentrations (<1 mol %), and henceforth, we refer to the gelation process as due to Fe^3+^ ions and refer solely to its bulk concentration. The “gel front” corresponds to the position of the sol-gel transition, i.e., opaque gel formation. In order to quantify the time evolution of these two fronts, the curves in Figure 2 are fitted to a Logistic function to evaluate the position and the front and its width:

(1) $$I\left(z\right)=\frac{I_{max}}{1+e^{-k(z-z_{front})}}+I_{min}$$

where I(z) is the mean pixel intensity, *z* is the coordinate of the front (see Figure 2b), $z_{0}$ is the midpoint of the curve, $I_{min}$ is the baseline intensity and *k* accounts for the steepness of the function. The front width *w* is obtained from $z_{I_{max}}$−$z_{I_{min}}$. As shown in Figure 2a, $z_{fast}$ is determined from the inflection in the sigmoidal curve towards the plateau at $I_{max}$.

**Figure 3:**
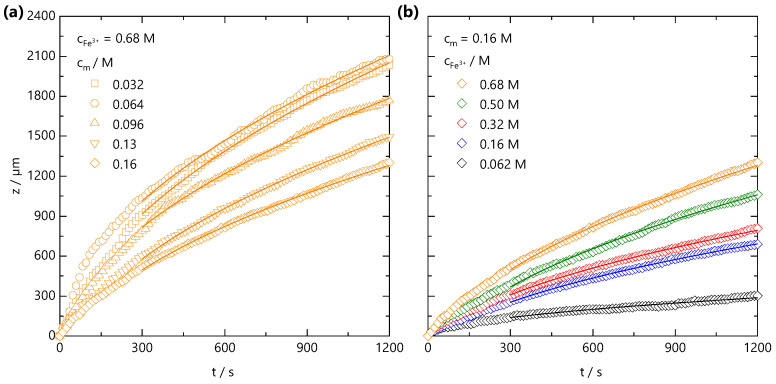
(**a**) Front position $z_{front}\left(t\right)$ measured at fixed ion concentration $c_{{\mathrm{Fe}}^{3+}}$ = 0.68 M, and varying polymer concentration, from $c_{m}$ = 0.032 to 0.16 M. (**b**) Front position $z_{front}\left(t\right)$ measured at fixed $c_{m}$ = 0.16 M and varying ion concentration $c_{{\mathrm{Fe}}^{3+}}$ = 0.062–0.68 M. The lines are data fits to $z\left(t\right)={\left(D_{f}t\right)}^{1/2}$ for t>300 s, where $D_{f}$ is a descriptive 1D diffusion coefficient. Non-Fickian deviations at short timescales are attributed to inertial effects during introduction of the salt solution to the confined NaCMC solution.

Both the fast front and the gel front grow with the square root of the time, as seen in Figure 2c,e, expected for 1D processes dominated by Fickian diffusion [42]. The influence of polymer and added salt concentration on the front propagation kinetics are examined in Figure 3a,b, respectively. At relatively short timescales, deviations from the $t^{1/2}$ power law are observed, which appear both NaCMC- and Fe^3+^-concentration-independent. In other frontal processes, an induction time is often required for the front to reach a “steady-state” [37], and we attribute this deviation to inertial effects associated with the introduction of the salt solution to the confined NaCMC solution. After sufficient time (≲300 s), fronts exhibit a $t^{1/2}$ scaling for all the polymer and salt concentrations studied, and a linear relationship between the front position and the square root of the time is observed, as shown in Figure S1.

**Figure 4:**
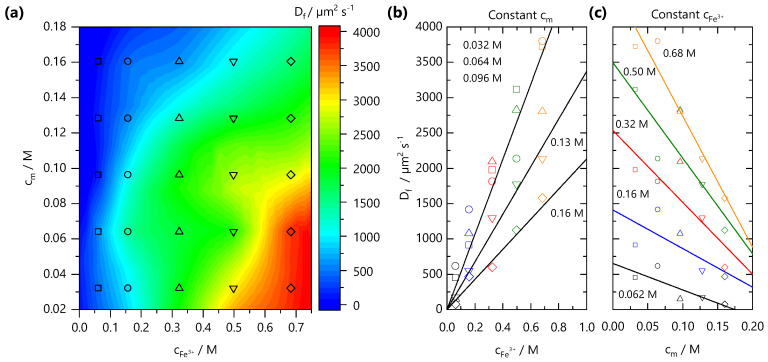
(**a**) Effective front diffusion coefficients $D_{f}$ as a function of polymer $c_{m}$ and ion $c_{{\mathrm{Fe}}^{3+}}$ concentration, plotted as a 2D contour map. The markers indicate experimental data points. (**b**) Dependence of $D_{f}$ with ion concentration $c_{{\mathrm{Fe}}^{3+}}$, at fixed $c_{m}$. (**c**) Dependence of $D_{f}$ with polymer concentration $c_{m}$ at fixed ion concentration $c_{{\mathrm{Fe}}^{3+}}$. Lines are guides to the eye.

From front kinetic data fits $z\left(t\right)={\left(D_{f}t\right)}^{1/2}$, we extract $D_{f}\left(c_{m},c_{{\mathrm{Fe}}^{3+}}\right)$ values which are shown as both a 2D contour map, in Figure 4a, and slices at fixed $c_{m}$ and $c_{{\mathrm{Fe}}^{3+}}$ in Figure 4b,c, respectively. Two trends are clear: increasing $c_{{\mathrm{Fe}}^{3+}}$ yields faster fronts, and the increasing $c_{m}$ yields slower fronts. Similar observations have been made in the formation of Ca-alginate gels [43,44] and divalent cation-polygalacturonate gels [45]. A greater concentration gradient of mobile cations increases the flux of ions and front propagation whilst increasing $c_{m}$, provides a larger number of binding sites for Fe^3+^ ions and thus consumes more Fe^3+^ ions during front propagation. This is combined with the hindrance of ion diffusion through the gel formed slowing the front propagation kinetics. It is conceivable that charge inversion of the polyelectrolyte during crosslinking could be dominant in slowing ion diffusion through the matrix. The polyelectrolyte solution viscosity is also expected to play a role in setting the front kinetics. Through comparison of the NaCMC solution viscosity (previously reported in Ref. [11]) with the experimentally measured $D_{f}$ values, shown in Supplementary Information Figure S2, we find that the viscosity changes by ∼2 orders of magnitude between the lowest and highest $c_{m}$ studied here, whilst the $D_{f}$ varies by less than 1 order of magnitude, at a fixed $c_{{\mathrm{Fe}}^{3+}}$. Evidently, the effective $D_{f}$ subsumes a number of contributions, and is not dominated by the polyelectrolyte solution viscosity.

Braschler et al. [44] formulated a framework to describe gelation fronts in Ca-alginate systems in the context of reaction-diffusion theory, building on the Mikkelsen–Elgsaeter model [46]. They defined a single, dimensionless parameter λ to describe the transport mode of the polyelectrolyte undergoing gelation and, for our system:

(2) $$\lambda =\frac{D_{f}}{D_{NaCMC}}$$

where $D_{f}$ is the effective diffusion coefficient of the front and $D_{NaCMC}$ is the diffusion coefficient of NaCMC. The effective diffusion coefficient describes the movement of the gel front, and incorporates the influence of various factors such as changes in the local viscosity experienced by ions and polymer–ion interactions. $D_{f}$ is therefore not equivalent to the diffusion coefficient of the Fe^3+^ ions. Taking the range of values of $D_{f}$ determined in this work, and $D_{NaCMC}$ of a similar $M_{w}$ NaCMC from Sitaramaiah and Goring [47], we approximate λ≃ 7–290 for the NaCMC and Fe^3+^ concentration ranges investigated here. In the theoretical framework of Braschler et al., λ≫1 is indicative of a network formation regime where the polyelectrolyte does not diffuse significantly before quasi-instantaneously reacting with the diffusing ion, yielding a narrow depletion zone and thus a relatively constant polyelectrolyte concentration across both the gel zone and the adjacent polyelectrolyte solution. This behavior contrasts with, for instance, Ca-alginate gelation, where λ<1 and a depletion zone appears ahead of the gelation front. We would therefore expect the gels generated with this system to be much more spatially homogeneous than those of Ca-alginate. Léger et al. provide a detailed theoretical analysis of systems with only one component that can diffuse, and show that the front propagation follows the classical 1D diffusion equation [48]. Following either Braschler’s or Léger’s analysis here would not add more insight given that the front position and $D_{f}$ values we extract will largely reflect the diffusion of Fe^3+^ ions during the gelation.

### 2.2. Molecular Interactions between NaCMC and Fe Ions

**Figure 5:**
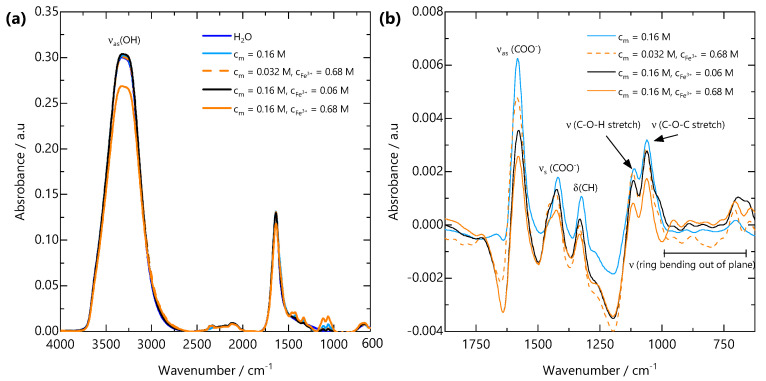
(**a**) FTIR spectra from H_2_O, NaCMC solution and FeCMC gels over the wavenumber range 4000–600 cm^−1^. All spectra are dominated by the asymmetric OH stretching band $\nu_{as}$(OH) from H_2_O. (**b**) H_2_O-subtracted spectra over the wavenumber range 1875–625 cm^−1^. Upon addition of Fe^3+^, the asymmetric carboxylate stretching band $\nu_{as}$(COO^−^) at 1582 cm^−1^ shifts to lower wavenumbers, the symmetric carboxylate stretching band $\nu_{s}$(COO^−^) at 1419 cm^−1^ shifts to higher wavenumbers and a shoulder develops. These peak shifts are indicative of bidentate binding of carboxylate groups to Fe^3+^ ions.

FTIR spectral features were analysed to elucidate the interactions between the NaCMC and Fe^3+^ ions. The infrared absorption bands of NaCMC and its complexes with Fe are shown in Figure 5a. The appearance of a broad hydroxyl (OH) band at 3500–3000 cm^−1^ with approximately the same intensity is indicative of the presence of the same concentration of water in all the samples. This confirms that the gelation of NaCMC with Fe^3+^ does not involve discernible amounts of water expulsion from the gel, i.e., no syneresis [40]. To further analyse the spectra, in the region where the carboxylate vibrations are expected, the water spectrum was subtracted from all spectra, and the resulting spectra are shown in Figure 5b. The region displays two major peaks arising from the asymmetric $\nu_{as}$(COO^−^) and symmetric $\nu_{s}$(COO^−^) stretches of the carboxylate groups at 1582 and 1419 cm^−1^, respectively. Since the interaction between the metal cation and the coordinating carboxylate group directly affects electronic delocalisation and consequently the stretching frequencies of the carboxylate ion, the position and shape of these peaks provide insight into the binding between COO^−^ and Fe^3+^. The difference between the asymmetric and symmetric stretches for the carboxylate ion Δν(COO^−^) = $\nu_{as}$(COO^−^)–$\nu_{s}$(COO^−^) has been shown to reflect the coordination mode of the carboxylate group to a metal cation [49,50]. Studies conducted on metal acetates suggest that a separation of 105–140 cm^−1^ is associated with monodentate bonding, 145–185 cm^−1^ is associated with bidentate chelate bonding and that 180–190 cm^−1^ is indicative of bidentate bridging between two metal cations [50]. These separations have been shown to hold for carboxylate-containing polyelectrolytes, including alginate and NaCMC [51,52].

Based on the difference between the symmetric and asymmetric carboxylate absorption bands for pure carboxylate-containing polyelectrolyte solutions and for those with added multivalent cations, the binding configuration could be summarized as follows:

- If there is an absorption band corresponding to carbonyl (C=O) groups in the spectrum and Δν(COO${}_{complex}^{-}$) >Δν(COO${}_{pure}^{-}$), monodentate bonding is prevalent.
- If there is no absorption band corresponding to carbonyl (C=O) groups in the spectrum and Δν(COO${}_{complex}^{-}$) <Δν(COO${}_{pure}^{-}$), bidentate chelation of the metal cation is prevalent.
- If there is no absorption band corresponding to carbonyl (C=O) groups in the spectrum and Δν(COO${}_{complex}^{-}$) ≈Δν(COO${}_{pure}^{-}$), bidentate bridging of two metal cations is prevalent.

The FTIR spectrum of FeCMC complexes in Figure 5b shows an absence of a carbonyl absorption band (at ∼1750 cm^−1^). Table 1 shows the NaCMC and Fe^3+^ concentrations for solutions and gels measured by FTIR, their carboxylate asymmetric and symmetric stretching frequencies and the difference between them. All Δν(COO^−^) values are indicative of bidentate chelation of the counterion Na^+^ or Fe^3+^ cation. The Δν(COO^−^) values decrease upon complexation of Fe^3+^ and with increasing $c_{m}$ and $c_{{\mathrm{Fe}}^{3+}}$. These results are consistent with previous reports on the interaction of NaCMC and Fe^3+^ [34,53]. Given the bidentate complexation of Fe^3+^ by COO^−^ and the presence of a sol-gel transition, we infer that multiple COO^−^ groups must act as crosslinking points. Further, noticeable shifts in the lower wavenumber region corresponding to out-of-plane ring bending (625–1000 cm^−1^) suggests distortion of the glycosidic ring at the length scale of a single monomer. Variations in characteristic vibrational modes of polyelectrolyte-multivalent salt complexes provides useful insights into their bond orientation and molecular binding mechanism. This FTIR-based assessment of ion binding lays the molecular framework for understanding the spatial evolution of the gel structure at larger length scales.

### 2.3. Spatio-Temporal Evolution of the Gelation Front Examined by SANS

**Figure 6:**
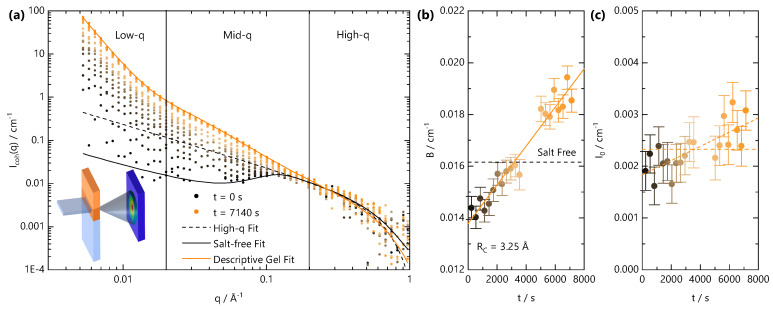
Travelling front small angle neutron scattering (SANS) experiment, carried out with a stationary cell-beam slit arrangement, while the gelation front traverses the illuminated volume by the neutron beam. (**a**) Schematic of the experimental setup (inset) and time-resolved scattering intensity for a sample with $c_{m}$ = 0.13 M and $c_{{\mathrm{Fe}}^{3+}}$ = 0.68 M acquired over 7140 s at selected time intervals (accumulated over 300 s). Lines are fits to Equation (8) (dashed black line) and Equation (6) (solid black line) to the salt-free data at *t* = 0 and Equation (11) (solid orange line) to data at *t* = 7140 s. (**b**) Background values extracted from fits to Equation (8) of the SANS profiles in (**a**). Dashed line is the background scattering value for salt-free solution. (**c**) $I_{0}$ values extracted from fits to Equation (8) of the SANS profiles in (**a**). Dashed lines indicate that, within error, both a constant I(0) and a linearly increasing $I_{0}$ value are consistent with the data.

We next examine the gel formation process at the nanoscale using time-resolved SANS during front propagation. The neutron beam slit is positioned 3 mm below the initial solution meniscus, and the cation solution is carefully added to initiate the gelation front. Given the weak scattering signal of the system, SANS data are accumulated in 300 s intervals. Figure 6a shows a schematic of the experiment, alongside the time-resolved coherent scattering curves. The scattering profile at t=0 s is characteristic of a salt-free semi-dilute polyelectrolyte with a characteristic broad peak centred at $q^{*}=0.12$ Å^−1^ and yields a correlation length of ξ=52 Å in agreement with the previously established concentration dependence of ξ for this NaCMC sample [29,31]. Equation (6) describes the data well over the entire *q*-range. Over time, as the front traverses through the volume illuminated by the neutron beam slit, the scattering profiles shift. First, the mid- and low-*q* scattering ($q<q^{*}$) increase and cause a disappearance of the broad polyelectrolyte correlation peak. The intensity increases further with time, and at long times (>5000 s), three clear structural features can be identified in the low-, mid- and high-*q* regions. To these data, Equation (11) provides an adequate and instructive, albeit empirical fit. At *t* = 7140 s, the apparent correlation length ξ=40 Å reaches an asymptotic value with an exponent m>2, which is indicative of a reduction in the polymer chain dimensions; *m* never reaches values of 3, which would be indicative of a chain collapse. The time-dependent parameters from fits to Equation (11) are given in Figure S3.

Given the temporal dependence of the sol-gel transition within the illuminated scattering window, careful subtraction of the “background” (*B*), which arises primarily from incoherent scattering, is essential. Data analysis is rendered complex by the fact the front propagation within the CMC solution is expected to involve multiple diffusion processes; the salt, D_2_O and polyelectrolyte (albeit relatively much slower) can diffuse with different rates and inherently have different scattering cross-sections. Further, given the weakly scattering coherent signal of the polyelectrolyte and the spatial and temporal averaging of the measurements, a precise decoupling of scattering contributions is challenging. A “conventional” data treatment, by fixing the background from the NaCMC concentration and incoherent contribution $S_{inc,NaCMC}=0.60\pm 0.05$ cm^−1^, yields unphysical features of the scattering at high *q*, such as an apparent chain cross sectional radius $R_{c}$ smaller than a single carbon-carbon bond. We therefore fit the high-*q* region to Equation (8) with a fixed $R_{C}=3.25$ Å with the background as a free parameter. The fitted *B* and I_0_ values are given in Figure 6b,c, respectively. After an initial decrease from the expected salt-free *B* value, calculated by consideration of $S_{inc,NaCMC}$, water content (15 ± 4% *v*/*v*) of the polyelectrolyte and volume fraction in solution, the fitted *B* was found to linearly increase with time as the gelation front evolved. This is consistent with the addition of Fe^3+^ solution into the neutron beam footprint over time, which has a largely flat scattering signal with $I_{{\mathrm{Fe}}^{3+}}≃0.01$ cm^−1^.

Given the effective diffusion coefficient determined for the NaCMC and Fe^3+^ concentration investigated here, $D_{f}=2130\;$ m^2^ s^−1^, the front is expected to enter the beam footprint at t=1440 s and move past at t∼8480 s and so the background is expected to change monotonically within the experimental time. The SANS profiles clearly change prior to the expected arrival of the front within the illuminated beam volume, and this can be attributed to (i) additional hydrostatic pressure driving faster diffusion than our microchannel experiments would predict; from the time dependence of the SANS profile, we estimate an upper bound for $D_{f,vertical}$ to be ∼5700 m^2^ s^−1^; (ii) contribution from the fast front $z_{fast}$ (Figure 2) of Fe^3+^ ions, which do not crosslink sufficient to yield an optically opaque gel and diffuse with $D_{f,fast}>$ 5700 m^2^ s^−1^. From our previous measurements of NaCMC with divalent ions [31], small added salt concentrations cause significant changes in the SANS profiles. The two reasons are not mutually exclusive given that we would expect the fast front to also increase with hydrostatic pressure and to precede the gelation front.

In Figure 6c, $I_{0}\approx 0.0019$ cm^−1^ Å^−1^ at *t* = 0 s, which we independently calculate to be $c_{m}=0.13$ M, our known added NaCMC concentration. Over time, $I_{0}$ increases as a result of Fe^3+^ diffusion into the illuminated beam volume and complexation with NaCMC. Assuming that Fe^3+^ “instantaneously” reacts with NaCMC and stoichiometrically replaces the counterion, the $I_{0}$ value can be used to calculate the concentration of (now FeCMC) chains in solution. From the large uncertainty in the scattering at high *q*, both an increase in $I_{0}$ or a constant $I_{0}$ value are consistent with the data. The former yields an increase in $c_{m}$ to 0.2 M at ∼8000 s, a ∼50% increase in concentration, whilst the latter implies a negligible change in concentration associated with the shift in neutron contrast upon binding of Fe^3+^. A 50% change in concentration appears unlikely, given that the FTIR measurements confirm that syneresis does not occur, but we cannot exclude that a shift in the partial molar volume occurs upon gel formation, which increases the high-*q* scattering signal for a fixed concentration (mass by volume) of chains. An estimation of the fraction of laterally aggregated chains from the $I_{0}$ parameter is in principle possible [28], but without knowledge of the partial molar volume, the calculation becomes highly imprecise. Due to the insolubility of FeCMC in water, the determination of the partial molar volume is not possible using a capillary densitometer.

We also considered other, more common models for fitting gels, e.g., the Gauss–Lorentz model for gels from neutral flexible polymers [54,55], which we found did not fit the time-resolved data well. Specifically, the Gauss–Lorentz model did not capture the scattering from the chain cross-section at high *q*. Other models previously used to fit scattering from biopolymer solutions with added salt [31,56] (Equation (10)) capture the high-*q* scattering well but did not give good agreement over the mid-*q* region or the transition region to the low-*q* power law scattering. We also explored the feasibility of fitting the Beaucage model [57], which describes fractal objects with multiple length scales of structural features; while good agreement with the data could be obtained, these required 3 levels and 12 parameters, and many parameters appeared unphysical. Other authors, including Beaucage, have rightly discussed the suitability of this model in fitting SANS data [57,58], and therefore we chose not to analyse our data with this model. We have thus opted to use a descriptive model (Equation (11)), as, firstly, it gives good agreement with the data for t>5000 s and yields physically meaningful parameters.

**Figure 7:**
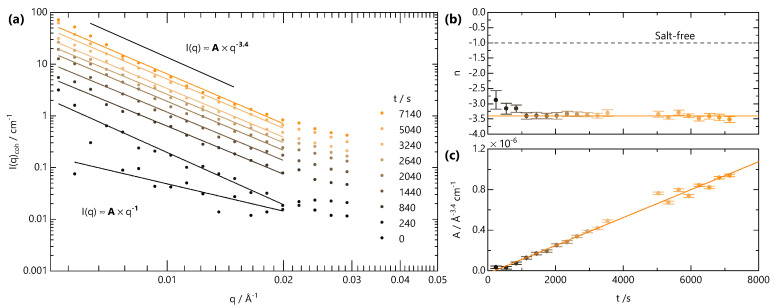
Analysis of SANS data for q<0.03 Å${}^{-1}$, in Figure 6. (**a**) The low-*q* intensity is fitted to a power law $Aq^{n}$. The exponent *n* and prefactor *A* to this power-law are plotted in panels (**b**,**c**), respectively. The exponent is determined by fitting *n* as a free parameter, while the *A* values shown were subsequently obtained by fixing the value of *n* as shown by the solid orange line in (**b**).

Finally, we analyse the low-*q* scattering separately with a power law to track the temporal evolution of the large-scale network structure, associated with crosslinking, in Figure 7. Initially, both *A* and *n* are used as fitting parameters. The variation in *n* from this approach is shown in Figure 7b. Starting from a value of n∼1, characteristic of fully dissolved NaCMC, *n* rapidly drops to a value of ∼3.4 before reaching a plateau. This value is indicative of a rough interface in a two-phase system, which likely arises from the clusters formed upon crosslinking. We therefore fix the value of *n* for and refit to yield the variation of *A* with time, as shown in Figure 7c. Prefactor *A* is found to increase linearly with time for t>540 s, much like the linear increase of *B* and $I_{0}$. In contrast to the front position, which depends on $t^{1/2}$, the gel structure appears to evolve with linear kinetics.

### 2.4. SANS Scanning Across a Quasi-Static Front Profile

**Figure 8:**
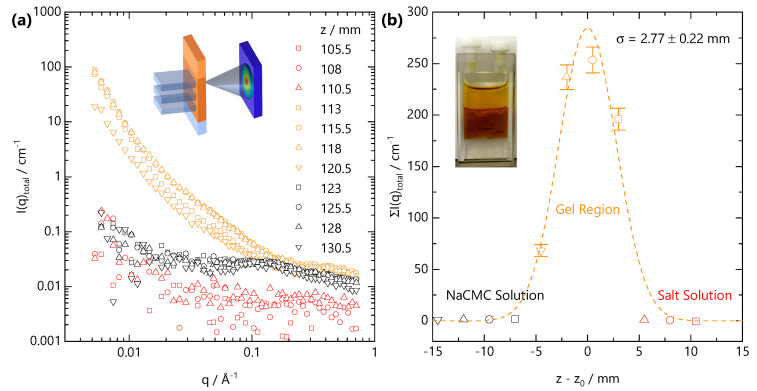
(**a**) Representative scattering profiles obtained from a vertical scanning SANS experiment, schematically shown in inset, across the gelation front. The sample corresponds to the same front propagation experiment reported in Figure 6 ($c_{m}=0.13$ M, $c_{{\mathrm{Fe}}^{3+}}=0.68$ M), and scanning was carried out under quasi-static conditions (see text) at long propagation time (*t* = 6.5 h) in 2.5 mm spatial intervals, identical to the vertical dimension of the beam slit. (**b**) Summed scattering intensity $\Sigma I{\left(q\right)}_{total}$ at each relative position to the initial front interface $z-z_{0}$; the profile can be described by a Gaussian profile centred on the initial front interface with σ=2.8±0.2 mm width.

After long times, selected here as ∼6.5 h following the onset of front propagation, the front slows down sufficiently such that it can be considered quasi-static. Under these conditions, SANS acquisition times become shorter than the timescales for front propagation, and the front profile can be spatially mapped with the neutron beam slit. Figure 8a shows the scattering intensity along the vertical position *z*, measured in 2.5 mm steps vertically along the cuvette. The initial NaCMC-FeCl_3_ interface was located at position *z* = 113 mm at the onset of gelation. SANS profiles can be categorised according to their *q*-dependent scattering. Orange curves fall within the Fe-CMC gel region, black within the solution below the front and red curves within the salt solution above the front. Figure 8b shows the summation of the scattering intensity over the entire *q*-range as a function of the relative position $z-z_{0}$ from the initial interface. An optical image of the front prior to SANS scanning is included in the inset. The expected front position at this time is ≃7 mm below the initial interface. We resolve (4 × 2.5) mm vertical steps with scattering pattern indicative of gelation with one, closest to the NaCMC solution, with much lower intensity. This indicates that the front has actually travelled >7.5 mm and that the lowest intensity profile is likely spatially averaged over some salt-free solution as well as the gel. Our estimation for $D_{f,vertical}$ would over-predict the front distance (∼11.5 mm) but suggests that this value, given the spatial resolution of the beam (2.5 mm) and temporal resolution (300 s) in our front evolution experiment (Figure 6), corresponds to an upper bound.

Owing to the strong scattering from the gel and relatively weak scattering from both salt-free solution and salt solution we do not observe the sigmoidal front profiles that we see with optical microscopy (Figure 2). Instead the front profile resolved by SANS can be described by a Gaussian profile:

(3) $$g\left(z^{\prime }\right)=\frac{C}{\sigma \sqrt{2\pi }}\exp\left(-\frac{1}{2}{\left(\frac{\left(z^{\prime }-\mu \right)}{\sigma }\right)}^{2}\right)$$

where $z^{\prime }\equiv z-z_{0}$ is the the relative front position, μ is the centre of the distribution, $\sigma^{2}$ is the variance and *C* is a scale factor. An acceptable fit to the data in Figure 8b is obtained for μ=0.0±0.2 mm and σ=2.8±0.2 mm. We note that a Gaussian profile may not fully capture the front profile, which is likely asymmetric. These results indicate the front is centred on the initial position of the solution–salt interface, with some back-swelling of the polymer network into the salt solution, likely due to 5 mm path length of the cuvette which cannot be approximated to a “1D geometry”. The variance is, within uncertainty, the same as the vertical size of the beam window and the vertical step size and indicates that the front remains relatively sharp. The optically determined front width (w∼500 μm at *t* = 1200 s, Figure 2e) is approximately 1/5 of the neutron beam size and will be difficult to resolve with our neutron beam’s spatial resolution.

Both the SANS profiles and the summed intensities would suggest, in contrast to our optical measurements, that Fe^3+^ does not diffuse significantly ahead of the front, based on the unchanged SANS profiles in the adjacent NaCMC solution region. Small (mM) concentrations of added divalent salt have pronounced effects of the SANS profile of NaCMC solutions [31], and Fe^3+^ diffusion into the solution would have significantly altered these otherwise “salt-free” profiles. At z=120.5 mm, in the gel region, we likely spatially average over the fast front position and therefore do not observe any screening of the polyelectrolyte peak in the salt-free solution below. Within the gel-region, the summed intensity between the four measured positions varies significantly (∼50–250 cm^−1^), which could be interpreted in terms of spatial heterogeneity, or that the gelation process is non-ergodic. Secchi et al. [59] have previous shown that Ca-Alginate are dynamically heterogeneous and that heterogeneity depends on the local “age” of the gel. Evolution in scattering intensity as function of vertical position and therefore local “age” of the gel structure will likely reflect coarsening and rearrangement of crosslinks occurring within the gel. In our time-resolved experiments (Figure 6 and Figure 7), we observe an increase in predominantly the mid- to low-*q* scattering as the gel “ages”. The high-*q* scattering, reflecting the concentration of chains (Equation (8)), appears approximately constant (within 10%) and is therefore consistent with the expectation of λ≫1 from our optical measurements and that the front is sharp and propagates without a NaCMC depletion zone ahead of it.

### 2.5. Microfluidic Hydrogel Microparticle Formation

**Figure 9:**
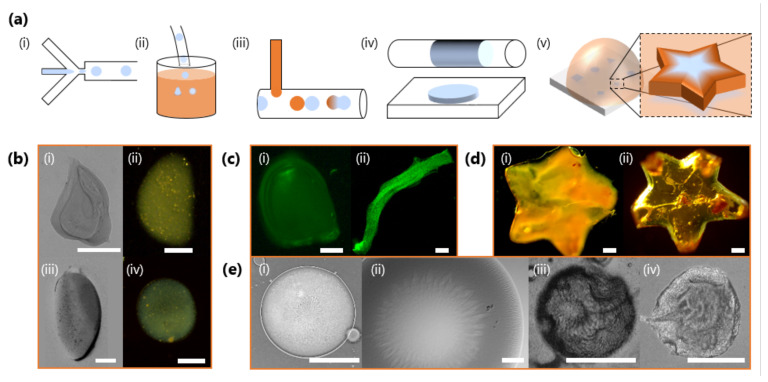
(**a**) Schematics of illustrative strategies to generate FeCMC hydrogel microparticles (HMPs) of varying morphologies. (i) Microfluidic droplet generation with a flow-focussing geometry. (ii) Ex situ immersion of NaCMC droplets into a salt solution reservoir to induce gelation. (iii) In situ addition of salt solution, here via a T-junction, and droplet coalescence to induce gelation. (iv) Geometrical and volumetric confinement in microfluidics can trivially template the droplet shape during gelation. (v) Geometric confinement can be imposed with mould arrays to impart complex shapes. (**b**) Particles generated by ex situ immersion into a salt solution reservoir with $c_{m}=0.032$ M and $c_{{\mathrm{Fe}}^{3+}}=0.06$ M (i–iii) and $c_{{\mathrm{Fe}}^{3+}}=0.68$ M (iv). (**c**) Fluorescent images of particles with encapsulated FITC-tagged melamine microparticles (∼1 μm), produced by selective droplet coalescence with salt solution in microfluidics. Tuning relative inlet flow rates and geometrical confinement allows “bullet”-shaped particles (i) and fibres to be produced (ii). (**d**) Star-shaped particles fabricated from ex situ geometrically confinement before gelation with $c_{m}=0.16$ and $c_{{\mathrm{Fe}}^{3+}}=0.06$ M (i) and $c_{{\mathrm{Fe}}^{3+}}=0.68$ M (ii). (**e**) Surface structure and wrinkling of particles, including characteristic flower-like patterns arising during gelation (i,ii); and surface buckling upon dehydration (iii,iv). All scale bars are 300 m except for (**e**) (i), which is 150 μm.

Equipped with the knowledge of how the gelation kinetics can be tuned through both NaCMC and Fe^3+^ concentration, we next demonstrate the controlled fabrication of HMPs with microfluidics. Figure 9 illustrates different morphologies and structures obtained by tuning gelation kinetics, flow and spatial confinement, as well as surface structures which spontaneously arise during gelation and drying. In a series of experiments, droplets are first generated with a microfluidic flow-focussing geometry (Figure 9a,i) under moderate (<100 L min^−1^) flow rates, and then immersed ex-situ into a FeCl_3_ reservoir (Figure 9a,ii) where the droplets initially stratify to the carrier phase (oleic acid) salt solution interface. As this carrier phase film separating the droplets from the salt solution below thins, the droplets transfer into the salt solution initiating the gelation process. NaCMC-containing droplets can also be gelled in situ by droplet fusion or interfacial contact with the salt solution within the microfluidic channels (Figure 9a,iii), and the resulting particle shape tuned by varying the droplet volume and imposing geometrical confinement within the channels (Figure 9a,iv). ex-situ geometric confinement within more complex shaped moulds (Figure 9a,v) can be used to impart greater shape control over the resulting, large, templated HMP arrays.

Figure 9b shows optical micrographs of HMPs obtained through ex situ immersion of droplets. By using a lower concentration of Fe^3+^ in the salt solution reservoir, the gelation kinetics can be slowed down to allow gravity, or convective flow, to deform the moving droplets in solution, prior to gelation arresting its shape (Figure 9b,i–iv) [60]. Higher Fe^3+^ concentrations and faster gelation kinetics largely result in spherical HMPs (Figure 9b,ii). In situ gelation and the opportunities offered by geometrical confinement relative to droplet size/volume, and deformation under flow, can yield a plethora of particle morphologies, including “bullets” or filaments (Figure 9c). Here, these are accessed by increasing the droplet volume, through NaCMC solution flow rate, to confine droplets as large plugs in the outlet tubing before introducing Fe^3+^ ions via a T-junction to induce gelation. We demonstrate the encapsulation ability of this ionic gelation approach by loading selected particles/fibres with 1 μm and fluorescently-labelled particles; see also Figure 9c. Figure 9d illustrates how, irrespective of $c_{{\mathrm{Fe}}^{3+}}$ and gelation kinetics, HMPS can be moulded into more complex shapes ex situ. The two star-shaped particles appear optically different, reflecting differences in stoichiometry and resulting cross-linking density. Finally, FeCMC HMPs generated by this approach can exhibit complex surface structure, which spontaneously arises during gelation (Figure 9e,i,ii) or through subsequent drying (Figure 9e,iii,iv), yielding further wrinkling and buckling.

## 3. Conclusions

We have examined the gelation of salt-free NaCMC solutions with trivalent iron cations (Fe^3+^), relevant for the precise design and formation of HMPs. Measuring front propagation kinetics in linear microchannels, we find that the gelation process can be rationalised by a reaction-diffusion wavefront framework. After a short period (≲300 s) following the contact between the polyelectrolyte and salt solutions, gelation fronts travel with a $t^{1/2}$ dependence characteristic of a diffusive process. We observe both an optically opaque front as well as a faint, weak-contrast “fast front”, which precedes the gelation front and which we attribute to Fe^3+^ diffusion in sufficiently low concentrations as to not induce gelation. We have established front propagation kinetics as function of both NaCMC ($c_{m}$ = 0.032–0.16 M) and Fe^3+^ ($c_{{\mathrm{Fe}}^{3+}}$ = 0.06–0.68 M) concentrations, finding effective diffusion coefficients ranging from $D_{f}≃$ 73 to 3800 m^2^ s^−1^. $D_{f}$ values increase with increasing $c_{\mathrm{Fe}}^{3+}$, owing to an increased concentration gradient of the mobile Fe^3+^ ions, and decrease with increasing $c_{m}$, which is attributed to more binding sites for Fe^3+^ and the hindered ion diffusion through the gel matrix. In the theoretical framework of Braschler et al. [44], a dimensionless parameter λ, which compares the front diffusion to the polyelectrolyte diffusion, is approximated to be ≫1 for this system at all conditions investigated. This implies that the Fe^3+^ gelation front propagation occurs in an in situ polymerisation regime, where NaCMC can be considered quasi-static.

We have probed the molecular-scale interactions of Fe^3+^ with the polyelectrolyte via ATR-FTIR measurements. FTIR shows that the gels contain approximately the same proportion of H_2_O as the solution, thus confirming that the gels do not undergo syneresis during formation. Analysis of the 2000–600 cm^−1^ region of the spectra indicates shifts in both the asymmetric and symmetric stretching bands of the NaCMC carboxylate group upon Fe^3+^ complexation. The difference in wavenumbers of these two bands is indicative of the coordination of the carboxylate group to the metal. Our results are consistent with bidentate chelation of Fe^3+^, in agreement with previous reports. Out-of plane ring bending is also observable, suggesting that complexation of Fe^3+^ locally distorts the monomer segments.

To complement the gelation front propagation studies, we performed SANS measurements to yield the temporal evolution of nanostructure during the gelation and subsequently, under quasi-static conditions, spatially resolved the gel structure. Initially, the characteristic salt-free polyelectrolyte scattering peak disappears as the low-*q* and mid-*q* scattering intensity increases. The low-*q* scattering can be described by a power law decay that decreases from the salt-free value (−1) to an asymptotic value (−3.4) as the Fe^3+^ reaches the NaCMC solution. The scattering prefactor *A* evolves linearly with time, suggesting that the gel structural evolution does not follow the diffusive kinetics observed for the front propagation. At long times, when *t*>5000 s, an empirical model combining a power law, a Lorentzian profile and a cylindrical cross-section can describe the data well, yielding an apparent reduction in the correlation length, from the salt-free value, while exponent *m* points suggests that the uncrosslinked chains have a mass fractal scaling beyond that of the theta condition for dissolved chains; i.e., the polyelectrolyte chain dimensions are smaller than their unperturbed values. The value of *m* never reaches a value of 3, which would suggest that the uncrosslinked chains collapse and adopt a globular conformation. Spatial profiling of the front under quasi-static conditions suggests that our determined $D_{f}$ value is likely a lower bound for the front position in this vertical geometry, where hydrostatic pressure can accelerate the front propagation, and we provide an estimate for the upper bound. The front profile can be described by a Gaussian distribution centred on the initial front position and with a variance approximately equal to the vertical dimensions of the beam and the vertical step size. For the measurements that do not spatially average some salt-free solution, we attain, within ∼10%, the same SANS profile and confirm that the front appears relatively sharp when observed with SANS. Differences in scattering patterns are largely observed in the mid- to low-*q* regions and suggest that any heterogeneities are on length scales much larger than a single monomer or chain cross-section.

Informed by the composition dependence of the front gelation kinetics, we demonstrate a range of morphologies of HMPs that can be readily fabricated by from NaCMC by Fe^3+^ addition. Facile procedures include the generation of droplets by microfluidic flow-focussing and subsequent immersion or fusion or flow segmentation with the salt-solution, both in situ and ex situ. The relative rate of gelation and droplet deformation, or spatial confinement, can be employed to impart prescribed particle shapes (spheres, platelets or fibres), with consequences for function. Templating of large particle arrays can also be performed ex-situ with elastomeric moulds to impart complex 3D particle shapes. Further, surface patterning and buckling can emerge spontaneously following gelation on drying of HMPs. Overall, we show that ionotropic gelation of NaCMC provides a remarkably versatile and powerful means to fabricate HMPs, with a variety of practical applications, and that considerable control over microparticle shape and properties can be exerted through fundamentally examining the front propagation mechanism and kinetics.

## 4. Materials and Methods

### 4.1. Materials

NaCMC with nominal $M_{w}$ = 2.5 × 10${}^{5}$ g mol^−1^ and D.S. = 1.15–1.35 was purchased from Sigma Aldrich, Gillingham, UK. $M_{w}$ and D.S. were previously evaluated to be $M_{w}≃2.4\times 10^{5}$ g/mol and D.S. ≃1.3 [30,61]. Deionised (DI) water was obtained from a CENTRA ELGA LabWater system, and heavy water (99.9% D) and FeCl_3_ (anhydrous, ≥99.99%) were purchased from Sigma Aldrich, Gillingham, UK. Solutions were prepared by dissolving NaCMC in DI water or D_2_O, with the aid of a roller mixer. The hygroscopicity of NaCMC and residual water content was accounted for in the concentrations reported herein. For the NaCMC studied here, thermal gravimetric analysis (TGA) measurements resolved a bulk water content of 10±3% [31], whilst SANS measurements indicated ≃15 ± 4% from the incoherent background with incoherent scattering contribution of S${}_{inc,NaCMC}=0.60\pm 0.05$ cm^−1^. The NaCMC concentrations investigated ranged from 10 to 50 g L^−1^ which, accounting for the residual water content, yield 8.54–42.7 g L^−1^. Equivalent molar concentrations of monomer ($c_{m}$) are obtained by dividing mass-by-volume concentrations by the molecular weight of a NaCMC monomer unit (≃266 g mol^−1^). Fe^3+^ ions were introduced in the form of FeCl_3_, which was dissolved *w*/*w* in DI water or heavy water. A concentration range of 1–10% was selected, yielding equivalent molar concentrations of ∼0.06–0.68 M.

### 4.2. Microchannel Front Propagation Experiments

The gel front propagation experiments were performed in a linear geometry, following our previous work in surfactant mesophase dissolution kinetics [62], using either fabricated microchannels or employing borosilicate glass capillaries (CM Scientific, Silsden, UK). Microchannels for front propagation experiments were fabricated by closed-face frontal photo-polymerization (FPP) of a thiol–ene copolymer (NOA 81, Norland Adhesives, Cranbury, NJ, USA) sandwiched between glass microscope slides of 75 × 25 mm dimensions (VWR, Lutterworth, UK), following previously published procedures [63,64,65,66]. The microchannel height was defined by spacers with prescribed thickness (500 μm). Lateral dimensions were defined by selective UV-A exposure through a photomask. The unexposed monomer was developed by flushing with ethanol and (sparingly) acetone, revealing the newly formed microchannels. An inlet port was drilled with a diamond tip drill bit on the top slide, and a nanoport (IDEX Health & Science, Cambridge, UK, N-333 NanoPort, 10–32 Coned) was attached, aided by rapid epoxy adhesive. The channel cross section was fixed at height = 500 μm and width = 2000 μm, with aspect ratio 1:4, thus approximating a “1D” geometry. The channels/capillaries were completely filled with NaCMC solution before 0.8 mL of salt solution was carefully placed at the extremity of the channel, initiating the propagation of a gelation front. Optical images were acquired with a CMOS camera (Basler AG, Ahrensburg, Germany, puA2500-14ucMIC) with a 0.17 fps frame rate attached to an optical microscope with infinity-corrected objective (Olympus, UK, MPlan 5X).

### 4.3. Microfluidic Droplet Generation and Monitoring

Polydimethylsiloxane (PDMS) microfluidic devices were fabricated by well-established soft lithography approaches [67]. A flow focusing device was fabricated comprising a cross-flow junction followed by a longer channel with dimensions 500 μm width and 120 μm height. Inlet and outlets consisted of fluorinated ethylene propylene tubing (Cole Parmer, St Neots, UK, FEP) directly inserted into the PDMS and further sealed with rapid epoxy adhesive. Aqueous NaCMC solutions were injected as the dispersed phase and oleic acid as the carrier phase with a syringe pump (Harvard Apparatus, Cambourne, UK, Pump 33 DDS) at total flow rates between 0.5 and 100 μL min^−1^, where the dispersed phase flow rate was maintained at a lower rate than the carrier phase to produce various droplet sizes. Outlet tubing was either immersed into a reservoir of FeCl_3_ solution at a prescribed concentration or connected to a T-junction (Kinesis, St Neots, UK, 0.57 μL swept volume) with FeCl_3_ solution injected orthogonal to the flow direction. Optical microscopy was performed on an inverted transmission microscope (Olympus, UK, IX71) with 4X and 10X infinity-corrected objectives attached to a CMOS camera (Basler AG, Ahrensburg, Germany, acA1300-200uC).

#### Ex-Situ Moulding and Gelation

Moulds to shape hydrogel particles were fabricated by first generating the desired 3D shapes with FPP of NOA 81 supported on glass slides [36,66]. The free-standing shapes were then replicated in PDMS to generate a mould with well-defined shapes. The mould was then filled with NaCMC solution, and any excess removed, before carefully adding FeCl_3_ solution to form the particles.

### 4.4. FTIR Spectroscopy

FTIR spectroscopy of NaCMC solutions and gels upon FeCl_3_ addition was performed on a Bruker Tensor 27 System with DTGS detector in Attenuated Total Reflection (ATR) mode using a diamond crystal (Bruker, Coventry, UK, PlatinumATR accessory). A cover was placed over the sample to prevent evaporation during the measurement. For each spectrum, 64 single beam scans were averaged with 4 cm^−1^ resolution in the range of 4000 to 600 cm^−1^. Results were examined in absorbance using OPUS 8.5 software (Bruker, Coventry, UK). Raw spectra were processed with a standard baseline correction. To obtain the spectra of NaCMC and FeCMC gels, the spectrum of pure water was subtracted from the baseline corrected spectra.

### 4.5. Small Angle Neutron Scattering

SANS measurements were carried out on the time-of-flight SANS2D diffractometer at ISIS pulsed neutron source (Oxfordshire, UK), with an incident wavelength range of 2–14 Å and wavevector (*q*) range of 0.0052–0.969 Å^−1^ achieved by two detectors at 2.4 and 4 m away from the sample. Quartz cells of 5 mm path length (Hellma Analytics, Jena, Germany, 404-QX, double stopper tanks, 3.5 mL volume) were employed, to ensure a sufficient scattering signal, given the relatively small coherent signal of ≃4% NaCMC solutions. The wide cells have an internal width of 18.5 mm, enabling the use of a neutron beam footprint of 15 mm × 2.5 mm (a horizontal slit machined in cadmium), and thus yielding an illuminated sample volume of ≃190 L. SANS experiments were started by carefully preloading the cell with a prescribed (2 mL) volume of NaCMC 4% solution, approximately half-filling the cell. One millilitre of salt solution was then carefully introduced into the cell to achieve a planar, flat, liquid–liquid interface between the NaCMC and salt solutions. The front position was recorded (defining time *t* = 0) and the time-resolved SANS acquisition triggered. Two series of experiments were carried out: (i) recording the travelling front at a fixed beam position (3 mm below the initial front interface) as a function of time, and (ii) spatially resolving the front profile at long diffusive times, when the front moves slowly with respect to SANS acquisition times, and can be thus considered quasi-static. Measurement intervals were fixed at 5 min (300 s) for the travelling front experiments (no data were acquired during the time window 3500–5000 s, due to the ISIS neutron beam instability during the kinetic measurements). In the spatially resolved measurements, vertical scanning position intervals were fixed at 2.5 mm, equivalent to the vertical dimension of the neutron beam illuminated area.

MANTID software was used to reduce and merge the data from both detectors [68]. The polychromatic neutron source was binned in 0.05 δλ/λ steps from 1.75 to 16.5 Å. 2D detector data were rebinned into 1D I(q) vs. *q* data in logarithmic δq/λ steps of 0.1. A correction factor of 1.1 was used to calibrate data acquired with a rectangular aperture, using the scattering intensity of salt-free NaCMC solutions (also acquired with a conventional circular aperture) as a secondary standard, likely due to machining tolerances and corresponding neutron flux.

#### Modelling Scattering Data

The coherent, elastic scattering from a solution of a polyelectrolyte and counterions dissolved in a single solvent can be written as:

(4) $$\begin{array}{c} I\left(q\right)=\rho [{\bar{b}}_{m}^{2}S_{mm}\left(q\right)+2{\bar{b}}_{m}{\bar{b}}_{c}S_{mc}\left(q\right)+{\bar{b}}_{c}^{2}S_{cc}\left(q\right)] \end{array}$$

(5) $$\begin{array}{c} I\left(q\right)\approx \rho {\bar{b}}_{m}^{2}S_{mm}\left(q\right) \end{array}$$

where $S_{ij}\left(q\right)$ is the Fourier transform of correlation function between scattering species *i* and *j*, known as a partial structure factor. The corresponding contrast in scattering lengths are ${\bar{b}}_{i}$ and ${\bar{b}}_{j}$, respectively, and ρ is the number of monomers per unit volume. The contrast term for monomers (*m*) and counterions (*c*) is given by ${\bar{b}}_{i}=b_{i}-b_{s}{\bar{V}}_{i}/{\bar{V}}_{s}$, where $b_{i}$ and $b_{s}$ are scattering lengths of monomer/counterion and solvent, respectively, and ${\bar{V}}_{i}$ and ${\bar{V}}_{s}$ are the corresponding partial molar volumes. When dissolved in deuterated water (D_2_O), the contrast arising from monovalent sodium counterions is ∼6% of the polyelectrolyte contrast and only ∼50% of counter-ions are condensed onto the chain [69]. Therefore the dominant contribution to the coherent scattering is the first term in Equation (4), allowing approximation of the coherent scattering by Equation (5).

For polyelectrolyte chains, long-range Coulombic forces induce intermolecular correlations, even well below the overlap concentration $c_{p}\ll$ c*). The scattering profile thus generally comprises a structure factor that is difficult to model analytically. Dobrynin’s scaling model predicts that in dilute salt-free solution, chains are rod-like [70]. Consequently, c* is extremely small compared to neutral polymers, and we often observe experimentally polyelectrolyte solutions in the semidilute regime [30]. The scattering profile of polyelectrolytes in salt-free solution displays a correlation peak at wavevector $q=q^{*}$, which is related to the correlation length $\xi ≃2\pi /q^{*}$, yielding a solution mesh size.

To fit the entire salt-free scattering data, we take the descriptive approach introduced by Lopez et al. [29], which combines a power law and a semi-empirical expression to model the broad polyelectrolyte peak and scattering from rigid rod-like objects at high-*q* ($>q^{*}$):

(6) $$I\left(q\right)=\frac{A}{q^{n}}+H\left(q\right)I_{high-q},$$

where H(q) is an empirical peak function

(7) $$H\left(q\right)=1-\exp\left(-{\left(qd\right)}^{m}-kq\right),$$

with no specific physical meaning is attributed to parameters d,m or *k*, and

(8) $$I{\left(q\right)}_{high-q}≃\frac{I_{0}}{q}P_{CS}\left(q\right),$$

where the form factor a cylindrical chain cross-section is modelled by:

(9) $$P_{CS}\left(q\right)≃{\left[2\frac{J_{1}\left(qR_{c}\right)}{qR_{c}}\right]}^{2}$$

where $J_{1}$ is a first-order Bessel function of the first kind and $R_{c}$ is the cross-sectional radius of the chain. $I_{0}$ is a constant related to the neutron contrast, chain concentration and monomer segment size, expressed as $I_{0}=\rho b_{m}^{2}\pi /b^{\prime }$, where $b^{\prime }$ is the effective monomer length $b^{\prime }=b/B$ and *B* is the stretching parameter (typically ≃1 for salt-free solutions).

In practice, Equation (8) was initially fitted to the scattering data for q>0.3 Å${}^{-1}$ and with $I_{0}$, $R_{C}$, and an additional background term (*B*), as free parameters. Given the uncorrelated scatter in the R${}_{C}$ values obtained, R${}_{C}$ was then fixed at an average value of 3.25 Å, consistent with our previous work [29], and the data refitted with $I_{0}$ and *B* as the only free parameters.

The presence of salt requires additional cross terms in Equation (4) and makes analytical solutions challenging. An alternative descriptive expression to fit the scattering profile of semiflexible polyelectrolytes (including biopolymers) in excess salt, and with addition of multivalent salts up to their respective phase boundaries [31], has been previously introduced by Horkay et al. [56]:

(10) $$I\left(q\right)≃\frac{A}{q^{n}}+\frac{I_{0}}{{\left[1+{\left(q\xi^{\prime }\right)}^{2}\right]}^{1/2}}P_{CS}\left(q\right)$$

where the first term describes the power law scattering from clusters and the second term from the polymer network. ξ’ represents a length scale that characterises the spatial distribution of semiflexible or rigid polyelectrolyte chains in solution [71,72]. It is a different quantity than $\xi \equiv 2\pi /q^{*}$ obtained from the position of the polyelectrolyte correlation peak, described by scaling theory. We adopt a similar descriptive approach to modelling the scattering upon gelation of NaCMC with Fe^3+^ ions. We take a linear combination of a power law, an empirical Lorentzian expression and Equation (8) to describe the scattering profiles:

(11) $$I\left(q\right)≃\frac{A}{q^{n}}+\frac{I_{0,Lorentz}}{\left[1+{\left(q\xi \right)}^{m}\right]}+\frac{I_{0,highq}}{q}P_{CS}\left(q\right)$$

where $I_{0,Lorentz}$ is a constant related to the contrast and concentration of scattering objects contributing to the concentration fluctuations described by that term and *m* is an exponent describing the fractal dimension associated with the concentration fluctuation spectrum (e.g., m=2 for a Gaussian distribution).

## Figures and Tables

**Table 1:** COO^−1^ asymmetric and symmetric stretching frequencies and peak difference from FTIR.

$c_{m}$/M	$c_{{\mathrm{Fe}}^{3+}}$/M	$\nu_{\mathrm{as}}$(COO^−^)/cm^−1^	$\nu_{s}$(COO^−^)/cm^−1^	Δν(COO^−^)/cm^−1^
0.16	0	1582	1419	163
0.032	0.68	1582	1423	159
0.16	0.06	1578	1425	153
0.16	0.68	1578	1426	152

## Data Availability

Data is available upon request. SANS data is available at DOI 10.5286/ISIS.E.RB1720083.

## Supplementary Materials

The following are available online at https://www.mdpi.com/article/10.3390/gels7020044/s1, Figure S1: (a) Salt-free NaCMC solution viscosity as a function of concentration ($c_{m}$). Closed circles are data taken from C. G. Lopez and W. Richtering, Cellulose, 2019, 26, 1517-1534. Lines are power law fits to the data with given exponents; 0.68, 1.5, 3.4. Open circles are interpolated points from the data for concentrations of NaCMC used in this study. Solutions viscosities vary by ∼ 2 orders of magnitude between the lowest and highest concentrations studied here. (b) Effective front diffusion coefficients $D_{f}$ at concentration Fe^3+^ concentrations $c_{Fe^{3+}}$ taken from main paper Figure 4. For any given $c_{Fe^{3+}}$, $D_{f}$ varies by < 1 order of magnitude between the highest and lowest $c_{m}$; Figure S2: Fitting parameters corresponding to the fits of main paper Equation 10 to the evolving front data. (a) $I_{0,Lorentz}$ (b) ξ and (c) *m*. For t<5000 s, $I_{0,Lorentz}$ and ξ appear unphysical and the model does not fit the data well. For t>5000 s, the values of $I_{0,Lorentz}$ and ξ tend to asymptotic values of 0.70 cm^−1^ and ξ. *m* increases over time indicating the chain dimensions decrease below their unperturbed value; Figure S3: (a) Salt-free NaCMC solution viscosity as a function of concentration ($c_{m}$). Closed circles are data taken from C. G. Lopez and W. Richtering, Cellulose, 2019, 26, 1517-1534. Lines are power law fits to the data with given exponents; 0.68, 1.5, 3.4. Open circles are interpolated points from the data for concentrations of NaCMC used in this study. Solutions viscosities vary by ∼ 2 orders of magnitude between the lowest and highest concentrations studied here. (b) Effective front diffusion coefficients $D_{f}$ at concentration Fe^3+^ concentrations $c_{Fe^{3+}}$ taken from main paper Figure 4. For any given $c_{Fe^{3+}}$, $D_{f}$ varies by < 1 order of magnitude between the highest and lowest $c_{m}$.

## Abbreviations

The following abbreviations are used in this manuscript:

HMP	Hydrogel microparticles
NaCMC	Sodium carboxymethyl cellulose
D.S.	Degree of substitution
SANS	Small-Angle Neutron Scattering
FTIR	Fourier Transform Infrared Spectroscopy
PDMS	Polydimethylsiloxane
NOA81	Norland Optical Adhesive 81 (Photoresist)
