# Supplementary material for: Ionotropic Gelation Fronts in Sodium Carboxymethyl Cellulose for Hydrogel Particle Formation
Source: Gels. 2021 Apr 12;7(2):44. doi: 10.3390/gels7020044 (PMC8167666; doi:10.3390/gels7020044)
Supplement: Supplementary file 1 [file gels-07-00044-s001.pdf]

Supplementary Information: Ionotropic Gelation Fronts in Sodium Carboxymethyl Cellulose for Hydrogel Particle Formation

William N. Sharratt,[*,†] Carlos G. Lopez,[‡] Miriam Sarkis,[†] Gunjan Tyagi,[†] Róisín O'Connell,[†] Sarah E. Rogers,[¶] and João T. Cabral[*,†]

†*Department of Chemical Engineering, Imperial College London, London SW7 2AZ, UK*

‡*Institute of Physical Chemistry, RWTH Aachen University, Landoltweg 2, 52056 Aachen, Germany*

¶*ISIS, Rutherford Appleton Laboratory, Harwell, Didcot, OX11 0QX, UK*

E-mail: w.sharratt16@imperial.ac.uk; j.cabral@imperial.ac.uk

Phone: +44 207 594 5571

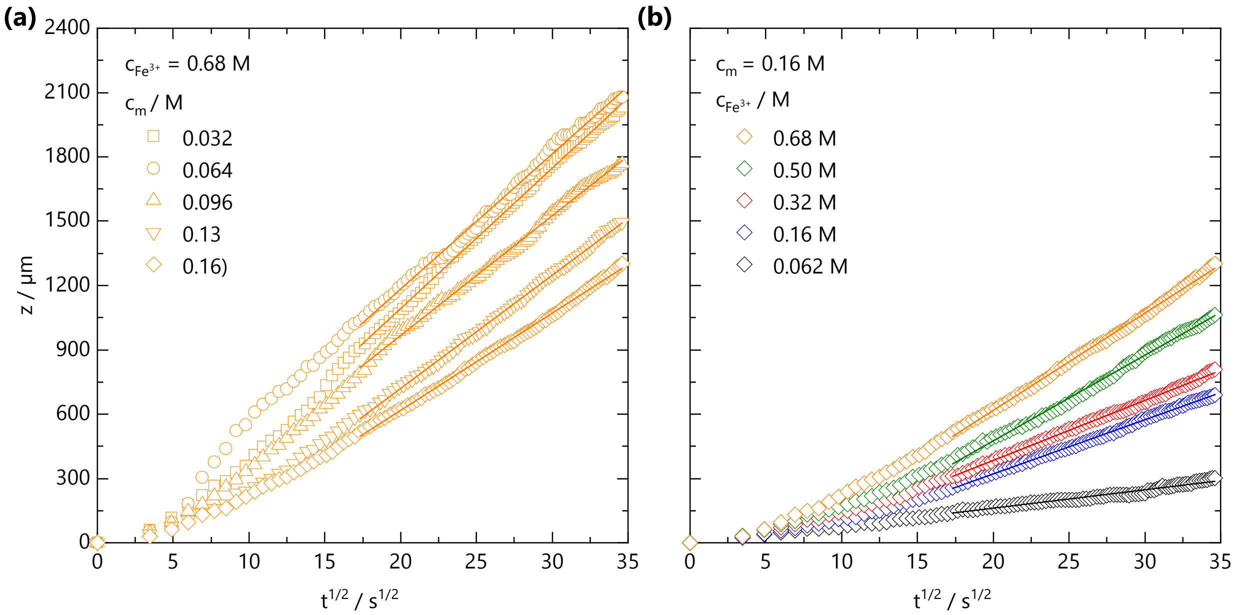

Figure 1: (a) Front position $z(t)$ against $t^{1/2}$ measured at fixed ion concentration $c_{Fe^{3+}} = 0.68$ M, and varying polymer concentration c_m = 0.032 - 0.16 M. (b) Front position $z(t)$ against $t^{1/2}$ measured at fixed polymer concentration c_m=0.16 M and varying ion concentration $c_{Fe^{3+}}$ = 0.062 - 0.68 M, and varying polymer concentration c_m = 0.032 - 0.16 M. The lines are linear fits for $t > 300$ s. The slope is equivalent to $D^{1/2}$ where D_f is a descriptive 1D diffusion coefficient.

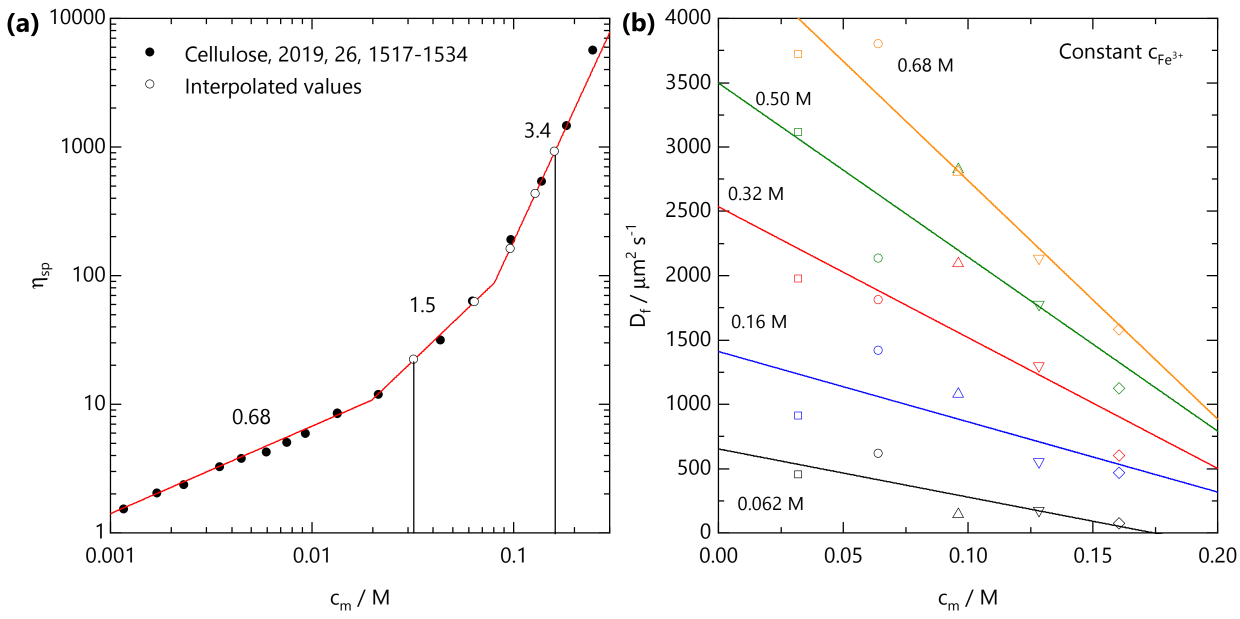

Figure 2: (a) Salt-free NaCMC solution viscosity as a function of concentration (c_m). Closed circles are data taken from C. G. Lopez and W. Richtering, Cellulose, 2019, 26, 1517-1534. Lines are power law fits to the data with given exponents; 0.68, 1.5, 3.4. Open circles are interpolated points from the data for concentrations of NaCMC used in this study. Solutions viscosities vary by $\sim$ 2 orders of magnitude between the lowest and highest concentrations studied here. (b) Effective front diffusion coefficients D_f at concentration Fe^{3+} concentrations $c_{Fe^{3+}}$ taken from main paper Fig. 4. For any given $c_{Fe^{3+}}$, D_f varies by < 1 order of magnitude between the highest and lowest c_m.

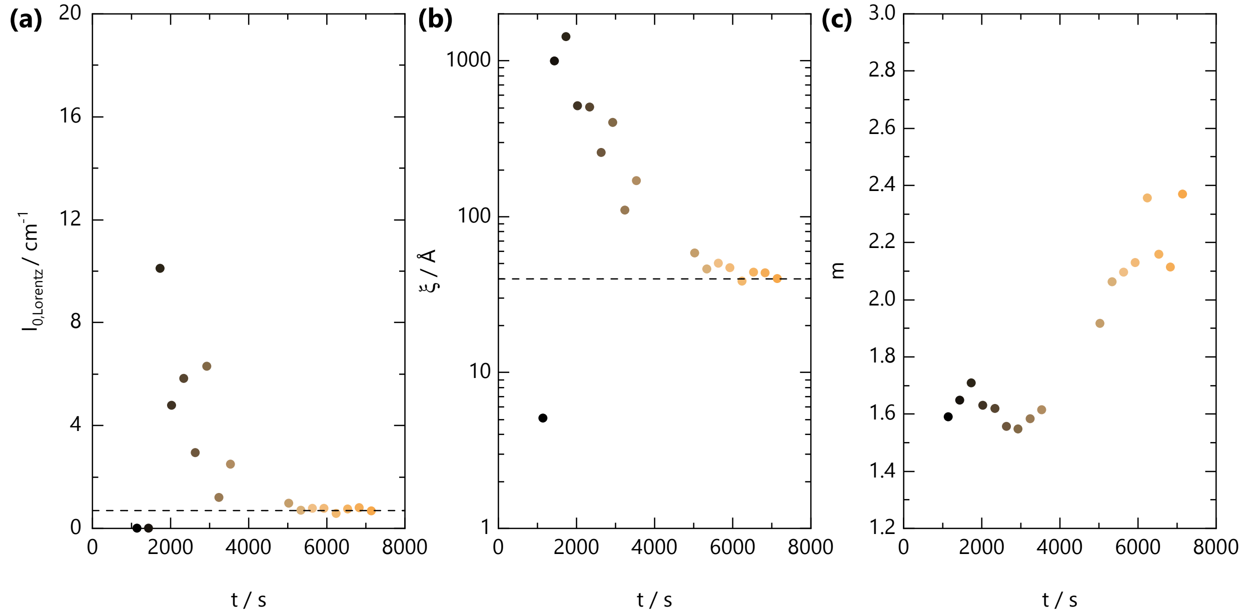

Figure 3: Fitting parameters corresponding to the fits of main paper Equation 10 to the evolving front data. (a) $I_{0,Lorentz}$ (b) ξ and (c) m. For $t < 5000$ s, $I_{0,Lorentz}$ and ξ appear unphysical and the model does not fit the data well. For $t > 5000$ s, the values of $I_{0,Lorentz}$ and ξ tend to asymptotic values of 0.70 cm^{-1} and ξ. m increases over time indicating the chain dimensions decrease below their unperturbed value.
